# Associations between Weight Status and Situational Motivation toward Fitness Testing in Physical Education: The Mediator Role of Physical Fitness

**DOI:** 10.3390/ijerph17134821

**Published:** 2020-07-04

**Authors:** Alberto Grao-Cruces, Alejandro Racero-García, David Sánchez-Oliva, David Blanco-Luengo, Alberto Nuviala, Tomás García-Calvo

**Affiliations:** 1 GALENO Research Group, Department of Physical Education, Faculty of Education Sciences, University of Cadiz, 11519 Puerto Real, Spain; racerogarciaalejandro@gmail.com (A.R.-G.); davidsanchez@unex.es (D.S.-O.); 2 Biomedical Research and Innovation Institute of Cadiz (INiBICA) Research Unit, 11510 Cádiz, Spain; 3 Faculty of Sport Science, University of Extremadura, 10003 Cáceres, Spain; tgarciac@unex.es; 4 Department of Sport and Computer Science, Pablo de Olavide University, 41013 Seville, Spain; dblalue@upo.es (D.B.-L.); anuvnuv@upo.es (A.N.)

**Keywords:** obesity, cardiorespiratory fitness, muscular fitness, mediating role, school, self-determination theory

## Abstract

Background: This article examines the differences in situational motivation toward fitness testing in physical education classes between non-overweight and overweight students, as well as the mediator effect of objective and perceived physical fitness on the relationship between weight status and motivation toward fitness testing. Methods: A total of 534 adolescents (298 boys, 55.80%) participated in the study. Perceived physical fitness and situational motivation toward fitness testing were measured through questionnaires, whereas weight status and physical fitness were objectively measured. Results: Overweight students had lower intrinsic motivation (*p* < 0.001), and higher external regulation (*p* < 0.01) and amotivation (*p* < 0.05) during fitness testing in a physical education class than their non-overweight peers. The influence of being overweight on motivation regulations toward fitness testing was mediated by objective physical fitness level for intrinsic motivation (*B* = −0.140), external regulation (*B* = 0.104) and amotivation (*B* = 0.146). Perceived physical fitness was also used as a second mediator between weight status and intrinsic motivation (*B* = −0.117). Conclusions: Strategies to improve objective and perceived physical fitness in overweight students are necessary to increase self-determined motivation during fitness testing in physical education lesson.

## 1. Introduction

Physical fitness (PF) refers to the ability to perform daily activities with vigour, as well as the demonstration of traits and capacities associated with low risk of premature development of the hypokinetic diseases [1]. Its assessment is a traditional component of school physical education (PE) in European and North American countries [2,3,4]. Fitness testing is a controversial element of the PE curriculum, whose presumably demotivating effect on students with low performance is a matter of concern [5,6]. This PE content has evolved in recent decades from a performance-based to a health-based approach [7]. Nowadays, the assessment of health-based PF components in PE, accompanied by an educational programme focused on health promotion [6,8], is encouraged in many countries [2,4].

In fact, the European Commission funded the development of evidence-based tests to assess health-related fitness feasibly in PE classes [9]. Cardiorespiratory and muscular fitness are priority components of this new battery of fitness tests, due to their predictive validity in young people [10]. These capacities are associated with a healthy lipid-metabolic profile, higher insulin sensitivity and glucose tolerance, lower blood pressure, higher bone density, healthy body composition, and higher cognitive and academic performance [11,12,13]. Therefore, it is important to detect low cardiorespiratory and muscular fitness levels of school-aged young people [13].

An aspect that has raised concern about students’ health-based PF levels is the progressive increase in overweight and obesity in young people in the last two decades [14]. Previous studies have shown that overweight and obesity have a negative influence on PF level; therefore, overweight students generally obtain worse PF test results than their non-overweight peers [15,16]. Likewise, this raises concern about the negative psychological consequences that fitness testing may have in overweight students [17,18]. Overweight students are sometimes mocked because of lower physical performance and suffer from discriminatory attitudes by their peers [19], and PE teachers [20]. However, to our knowledge, no empirical studies have examined overweight students’ motivational experiences during fitness testing sessions and how the objective PF level and perceived PF could influence these experiences.

Self-Determination Theory [21] provides a useful framework for the analysis of motivation associated with fitness testing in PE classes [5,6]. This macro-theory distinguishes different types of motivation ranging as a continuum from higher to lower self-determination levels. Intrinsic motivation represents the most self-determined motivation, and it refers to the pleasure and satisfaction derived from participating in a specific activity. The second block of motivation is extrinsic motivation, which is composed of integrated regulation (when students identify themselves with the importance of an activity and also integrate those identifications with other personal aspects), identified regulation (when students engage in an activity because they recognise and accept the underlying activity values or goals), introjected regulation (when students engage in an activity because they are pressured by self-esteem to act), and external regulation (when students participate in an activity because of external factors such as obtaining rewards, meeting external expectations, or avoiding punishments). Lastly, amotivation is the lowest level of self-determination, associated with students who are not motivated either intrinsically or extrinsically and, therefore, they have no intention of performing the activity [21]. According to Vallerand [22], these motivation regulations can appear on three hierarchically structured levels, which are: situational (e.g., fitness testing sessions), contextual (e.g., school PE classes), and global (personal predisposition to engage in activities with an intrinsic or extrinsic orientation). This theory supports the existence of three basic psychological needs, which act as mediators between social and personal factors and self-determined motivation [22,23]: autonomy (to be causal agents and to act in harmony with themselves), competence (to experience effectiveness in one’s pursuits), and relatedness (to have affectionate relationships with others). In this regard, numerous studies have shown that being satisfied with our perception of competence in a specific context or situation increases the most self-determined motivation [24]. In fitness testing sessions, students (consciously or unconsciously) often compare their competence with respect to themselves, their classmates or other criteria/data, thereby the psychological need of competence seems to be the most relevant factor. Consequently, following the proposals of the theory, perceived competence may have a clear mediating role between social and personal factors (i.e., weight status) and motivation.

Within this framework, Jaakkola et al. [5] found that students’ performance in PF tests is predictive of perceived sport competence, which has a positive effect on intrinsic motivation during fitness testing in PE classes. Although there are no more previous studies about it, this result indicates that objective and perceived PF could mediate a possible effect of being overweight on motivation toward fitness testing. This hypothesis is consistent with the Basic Psychological Needs Theory, included in the Self-Determination Theory [21,23].

The mentioned motivational theories are congruent with other theoretical frameworks, such as the Achievement Goals Theory [25], which proposes that the perception of competence in a specific situation modulates our cognitions and behaviours, from our individual beliefs. Likewise, Stodden et al. [26] explain the role that motor competence may have on the development of a healthy or unhealthy weight status. Health-related PF and perceived motor competence were suggested as mediating variables in the model. Later studies found that motor competence is both a precursor and a consequence of weight status and confirmed their inverse relationship across childhood and adolescence. In addition, evidence supports the mediating role of health-related PF and perceived competence between weight status and behavioural and motor variables [27].

**Figure 1:**
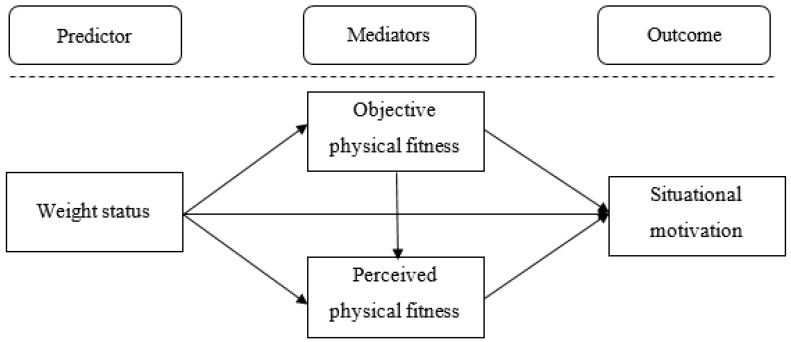
Multiple mediation model of the relationship between weight status and motivation during fitness testing in physical education with objective and perceived physical fitness as mediators.

Thus, to know the relationship between weight status and motivation during fitness testing, the direct and indirect effects shown in Figure 1 should be tested: (i) direct effect between weight status and situational motivation, (ii) mediated by objective PF, (iii) mediated by perceived PF, (iv) mediated by both consecutively.

The aim of the present study was double: to examine the differences in situational motivation toward fitness testing in PE classes between non-overweight and overweight students, and to analyse the mediator effect of objective and perceived PF on the relationship between weight status and situational motivation toward fitness testing in the PE context. We hypothesised that overweight students would show lower self-determined motivation in fitness testing classes and that PF mediates this relationship due to the negative influence of being overweight on objective PF, which would diminish the perceived PF and, consequently, generate less self-determined motivation.

## 2. Materials and Methods

### 2.1. Participants and Procedure

A total of 534 (298 boys, 55.80%) healthy Caucasian young people (12–16 years, 7th–10th grade) from four secondary schools of the Cadiz region (Southern Spain), previously selected by convenience sampling, participated in this cross-sectional study. For logistical reasons, the study was developed in the Spanish city of “El Puerto de Santa María”, a coastal city of 90,000 inhabitants in the Cadiz region. Culturally, thin girls and thin and muscular boys are popularised as body ideals in this region.

A total of six secondary schools of the mentioned city were invited to participate in this study through an invitation letter sent to the principals and physical education teachers. Four schools accepted the invitation. A meeting with the principals and physical education teachers in these centres was held to obtain the school’s agreement. Next, all adolescents from 7th to 10th grade were invited to participate. The parents of the adolescents received a brief flyer describing the study, including the inclusion criteria and an invitation to attend an informative meeting at the school. A meeting explaining the nature and objectives of the study was held in the four centres. Prior to the study, an informed consent document from the parents and adolescents was required. The study met the highest standards of safety and ethics, the laws of the country where it was performed, and the ethical standards established for this type of study by the Declaration of Helsinki. The study was approved by the Ethics Committees of the universities of the authors.

The data were collected between April and June 2017. The students were assessed in two PE classes of approximately 60 min during the same week. On the first day, the students completed the perceived PF questionnaire in their classroom, which took five minutes in average. Afterwards, their weight and height were measured. On the second day, the fitness testing class was held, during which the students performed the three tests included in the high-priority version of the PF ALPHA test battery. Some minutes later, they filled out the questionnaire examining their situational motivation toward the fitness testing class. Completing this questionnaire took five minutes in average.

### 2.2. Instruments

#### 2.2.1. Anthropometric Variables

Anthropometric measurements were performed on barefoot adolescents wearing light clothes. Weight was measured with an electronic scale [Type SECA 861; range, 0.05–130 kg; precision, 0.05 kg (SECA Ltd., Hamburg, Germany)], and height (in the Frankfort plane) was measured with a portable stadiometer [Type TANITA Leicester HR 001; range, 0–207 cm; precision, 1 mm (TANITA Corporation Inc., Arlington Heights, USA)]. We calculated their body mass index as weight/height squared (kg/m^2^). Overweight (including obesity) and non-overweight were calculated based on the international age-sex-specific body mass index cut-off [28].

#### 2.2.2. Physical Fitness

The cardiorespiratory and muscular fitness tests included in the PF ALPHA battery were used to assess PF [9]. Cardiorespiratory fitness was assessed by means of the 20 m shuttle run test. The participants were asked to run between two lines 20 m apart, while keeping the pace with audio signals emitted from a previously recorded compact disk (CD). The initial speed was 8.50 km/h, which was increased by 0.50 km/h per minute (1 min = 1 stage). The participants were instructed to run in a straight line, to pivot on completing the 20 m and to follow the pace in accordance with the audio signals. They were encouraged to keep running as long as possible throughout the course of the test. The test was over when the participant failed to reach the end of the lines concurrent with the audio signals on two consecutive occasions. Otherwise, the test ended when the participant stopped due to fatigue. All measurements were carried out under standardised conditions on an indoor rubber floor. The last stage completed was scored (precision of 0.5 stages).

Upper body muscular fitness was assessed by the Handgrip Strength Test, using a TKK5101 Grip D—class III dynamometer (Takey, Tokio, Japan) with 0.1 kg precision. The participants squeezed gradually and continuously for at least two seconds, standing during the whole test, with the arm straight and avoiding contact of any part of the body with the dynamometer with the exception of the hand being measured. Both hands were tested twice, alternately and choosing randomly the hand to be tested first. The sum of the best result obtained in each hand was used and, to account for differences in body size, it was expressed per kilogram of body weight [29]. Lower body muscular fitness was assessed by means of the standing long jump test. The participants stood behind a line with their feet together, and then pushed forward to jump as far as possible. The distance was measured from the take-off line to the back of the heel that landed closest to the take-off line. The test was repeated twice, and the best result (expressed in cm) was used for the subsequent statistical analysis [30,31].

A single muscular fitness z-score was calculated as the average of the two standardised scores from the muscular fitness tests (standing long jump and handgrip/weight). To generate the global PF variable, we calculated the mean of the cardiorespiratory and muscular fitness z-scores [29], both specific by age and gender.

#### 2.2.3. Perceived Physical Fitness

Perceived PF was assessed by using the physical condition dimension of the Spanish version of the physical self-perception profile [27], validated in young people and used in numerous studies with Spanish secondary education students [31,32]. The participants answered the question “When I engage in physical activity...”, followed by six items that make up the four-point scale, in which 1 means “completely disagree” and 4 means “completely agree”. This dimension showed an acceptable factorial validity through a one-factor-confirmatory factor analysis (CFA) (*CFI* = 0.956; *TLI* = 0.927; *RMSA* = 0.064; *SRMR* = 0.032) and good internal consistency (*Cronbach’s alpha* = 0.738) in this study.

#### 2.2.4. Situational Motivation

Motivation toward fitness testing in a PE context was assessed by using the Spanish version of the situational motivational scale (SIMS) [29]. The SIMS has been successfully used in previous studies for this situation in secondary education students [5]. The Spanish version consisted of 14 items aimed at assessing: intrinsic motivation (four items), identified regulation (three items), external regulation (three items), and amotivation (four items) in a specific situation. The participants answered the question “Why was I participating in this particular physical education class?”, to which they responded on a seven-pointscale between 1 (does not correspond at all) and 7 (totally corresponds). The factorial validity of this scale with this particular sample was demonstrated through a four-factor-CFA: *CFI* = 0.930; *TLI* = 0.910; *RMSA* = 0.061; *SRMR* = 0.060. The internal consistency of its dimensions was high (*Cronbach′s alpha* = 0.849, 0.761 and 0.765 for intrinsic motivation, identified regulation, and amotivation, respectively), except for external regulation (*Cronbach′s alpha* = 0.638), whose value, slightly lower than 0.700, can be considered acceptable due to the reduced number of items which comprise this dimension [33].

### 2.3. Data Analysis

Firstly, we calculated the percentages for categorical variables and means with standard deviation for continuous variables. Then, we analysed the correlations between global PF index, perceived PF and situational motivation by bivariate correlation analysis. Likewise, we examined the possible differences in these variables between overweight (including obese) and non-overweight students using an independent sample T-test. Finally, we analysed the total, direct and indirect effects of weight status (X) on situational motivation (Y) mediated by global PF index (M_1_) and perceived PF (M_2_) using multiple mediation analysis. In all mediation analyses, we used bootstrapping with 10,000 samples via the PROCESS procedure for SPSS [34], adjusted by age, gender and school. All analyses were performed using IBM SPSS Statistics v.21.0 for Windows (IBM Software Group, Armonk, NY, USA) and the level of statistical significance was set at *p =* 0.05.

## 3. Results

Table 1 shows some demographic information for all participants in addition to descriptive statistics for study measurements.

Table 2 presents bivariate correlations among the main study variables and differences by weight status. Objective and perceived PF were positively associated (*r* = 0.419, *p* < 0.001) and both were positively correlated with intrinsic motivation (*r* = 0.283, *p* < 0.001 and *r* = 0.365, *p* < 0.001 for objective and perceived PF, respectively) and identified regulation (*r* = 0.165, *p* < 0.001 and *r* = 0.299, *p* < 0.001 for objective and perceived PF, respectively), as well as negatively associated with amotivation (*r* = −0.199, *p* < 0.001 and *r* = −0.164, *p* < 0.001 for objective and perceived PF, respectively). Objective PF was also negatively associated with external regulation (*r* = −0.114, *p* < 0.01). Intrinsic motivation was positively associated with identified regulation (*r* = −0.713, *p* < 0.001) and negatively with external regulation (*r* = −0.172, *p* < 0.001) and amotivation (*r* = −0.433, *p* < 0.001). External regulation and amotivation were also positively associated (*r* = −0.309, *p* < 0.001). On the other hand, overweight students showed less objective and perceived PF (0.25 vs. −0.55, *p* < 0.001 and 2.89 vs. 2.68, *p* < 0.001, respectively), a lower intrinsic motivation (5.08 vs. 4.52, *p* < 0.001) and a higher external regulation and amotivation (4.62 vs. 4.83, *p* = 0.01 and 3.45 vs. 3.75, *p* < 0.05, respectively) than their non-overweight peers.

Table 3 shows the relationship between weight status and situational motivation regulations toward fitness testing in PE classes mediated by objective and perceived PF. Identified regulation was omitted, as it did not differ between non-overweight and overweight students. The total effect of being overweight on situational motivation was significant, negative for intrinsic motivation (*B* = −0.428, *95% CI* [−0.620, −0.235]) and positive for external regulation (*B* = 0.238, *95% CI* [0.076, 0.401]) and amotivation (*B* = 0.183, *95% CI* [0.093, 0.283]). The coefficient for the direct effect showed that weight status was not independently associated with intrinsic motivation (*B* = −0.180, *95% CI* [−0.382, 0.020]), external regulation (*B* = 0.145, *95% CI* [−0.034, 0.324]), or amotivation (*B* = 0.016, 95% *CI* [−0.191, 0.223]). However, the indirect effects of being overweight on situational motivation via objective PF were significant in all analysed cases, being negative for intrinsic motivation (*B* = −0.140, 95% *CI* [−0.242, −0.050]) and positive for external regulation (*B* = 0.104, *95% CI* [0.013, 0.198]) and amotivation (*B* = 0.146, *95% CI* [0.049, 0.252]). The results also showed an indirect effect of being overweight on intrinsic motivation mediated by objective PF and by perceived PF (*B* = −0.117, *95% CI* [−0.177, −0.069]). Moreover, contrast comparisons between these indirect effects (X→M_1_→Y vs. X→M_1_→M_2_→Y) indicated that they were not statistically different (*95% CI* [−0.145, 0.099]). No other indirect effect was significant. An inspection of the explained variance in each of the models showed that altogether weight status, objective and perceived PF (and covariates) accounted for 19.30, 3.60 and 4.80% of the variability in intrinsic motivation, external regulation and amotivation toward fitness testing in the PE lesson, respectively.

## 4. Discussion

This study examined the differences in situational motivation toward fitness testing in PE class between non-overweight and overweight students, as well as the mediatory role of objective and perceived PF on the relationship between weight status and motivation during fitness testing. The results showed that overweight students have lower self-determined motivation during fitness testing in PE class than their non-overweight peers. Likewise, the influence of weight status on situational motivation toward fitness testing is mediated by objective PF level.

In agreement with our hypothesis, overweight students showed lower intrinsic motivation and higher external regulation and amotivation toward fitness testing in PE class than their non-overweight peers. These results are coherent with the lower PF found among our overweight students compared to our non-overweight students, as reported in previous studies, which showed that being overweight impairs performance in PF tests [15,16]. These findings are also congruent with the model of Stodden et al. [26] and its revised version by Robinson et al. [27], where an unhealthy weight status is associated with a low physical fitness and a low motor competence. These results are also consistent with those reported by Lodewyk and Sullivan [18], who concluded that students who desire to be thinner are susceptible to lowered PF and motivation toward PF testing in PE classes. The identified regulation was not different between our overweight and non-overweight students. From a theoretical perspective, the concept of identified regulation refers to the recognition and acceptance of the underlying values or goals of an activity [21,35]. These aspects are less linked to the actual experience of an activity than to other self-determined motives (e.g., enjoyment or satisfaction), and we have no evidence that overweight students underrate the value of assessing health-related PF, thus this result was expected.

The lower self-determined motivation during fitness testing observed in our overweight students is in line with previous studies that showed concern about the experiences of overweight students in PE classes [36,37,38]. The complexity increases when adding that fitness testing is a controversial element of the PE curriculum, probably due to the fact, that in the past decades, PF and its assessment in PE did not have an educational approach to health promotion. Instead, performance-based physical fitness was tested to qualify and classify students according to their results in the fitness tests, which can be counterproductive in achieving educational objectives [7], especially among overweight students. Moreover, there are different sensitivities around the orientation that PE should adopt in order to be taught in the school (pro-health, pro-sport performance, pro-citizenship…) [1,39,40]. Notwithstanding the above, health-related PF is a compulsory content of PE in many countries, including Spain, where the control of health-based PF components in PE, accompanied by an educational programme focused on health promotion [6,8], is encouraged [2,4]. In fact, overweight students may be one of the groups that benefit the most from this health-based approach. Practical applications of studies such as this can be useful to make this control of health-related PF compatible with a self-determined motivation in both overweight and non-overweight students.

The significant relationships found between weight status and motivational regulations during fitness testing were not direct, but mediated by PF variables, in line with our hypothesis. We found a mediatory effect of objective PF, the only mediator, on the relationships of weight status with intrinsic motivation, external regulation and amotivation. These findings are consistent with the Basic Psychological Needs Theory [21,23], which highlights the mediatory role of psychological needs (the need to be competent in the current study) between social and personal antecedents (i.e., weight status) and motivation. The current study also confirms that objective PF level is a real indicator of competence during fitness testing in PE classes, and that the fulfilment of this psychological need is essential for self-determined motivation, which is in line with previous studies [41,42].

However, we expected to find a multiple mediatory model where being overweight diminished objective PF, which would influence perceived PF, which would in turn affects motivation in fitness testing classes. This double mediatory effect only occurred in the intrinsic motivation, and its strength did not differ from the model where objective PF was included as a single mediator. In this line, Jaakkola et al. [5] found that perceived sport competence was associated with intrinsic motivation toward fitness testing in PE, although the association with other situational motivation regulations was not significant. Therefore, perceived physical competence plays a relevant role in the relationship between objective PF and intrinsic motivation toward fitness testing. This is theoretically coherent, since the tenets of the Basic Psychological Needs Theory [21,23] suggest the need to be competent as a predictor of intrinsic motivation and the perceived PF as a subjective indicator of competence. In other words, those non-overweight students have higher objective PF levels, which in turn develop into better perceived PF, which contributes to feelings of enjoyment and satisfaction during fitness testing (intrinsic reasons). Nevertheless, this chain mediation effect of objective PF and perceived PF by weight status does not seem to determine other situational motivation regulations.

Weight status together with objective and perceived PF explained more than 19% of intrinsic motivation variance, a percentage noticeably higher than the 12% explained by the model of Jaakkola et al. [5]. However, our multiple mediation model could not explain 5% of the external regulation and amotivation variances, as reported by Jaakkola et al. [5].

## 5. Limitations, Strengths and Practical Applications

With respect to the limitations of this study, some of the questions used in the questionnaire may have been misinterpreted either deliberately or unintentionally by the participants, for example, due to fatigue after the fitness test. However, mistaken information was potentially minimised by the fact that the questionnaires were filled out anonymously, and all of them showed good reliability and validity for the sample studied. In addition, SIMS was the only questionnaire completed after the fitness test, for obvious reasons, and it was filled out after some minutes of physical rest. Another limitation of the present study was the non-inclusion of contextual motivation toward PE classes as a potential covariate. Although we initially included this variable, we decided to remove it, due to the low internal consistency found in three specific dimensions (Cronbach’s alpha < 0.650). However, we compared adjusted and non-adjusted results by contextual motivation, and insignificant changes were found. This is in line with the findings of Jaakkola et al. [5], who showed that the relationship between contextual motivation toward physical education and motivation in fitness testing sessions is low. Likewise, situational motivation could have conditioned the degree of effort reached during fitness testing and, consequently, the objective PF level. Although this bias has been assumed by other authors [5], in our study it affected a mediator (M_1_) and not the independent variable (X). Lastly, a mixed methods design might have increased the understanding of the relationship between weight status and motivation.

One of the main strengths of this paper was the inclusion of objective measures of weight status and PF. The PF variables were assessed with the PF ALPHA test battery, a recent evidence-based test battery created specifically for PE classes and recommended by the European Commission [9]. Moreover, this is the first study that examined overweight students’ experiences during fitness testing in PE classes. Another innovation of this study was the inclusion of multiple mediated analyses, in which we tested the mediatory role of objective and perceived PF in the relationship of weight status with motivation toward fitness testing sessions. Further research should examine teachers’ and overweight students’ perceptions during fitness testing by qualitative or mixed designs. This can be useful to develop methodological strategies to assess health-related PF in PE classes that are motivating for students, regardless of their weight status and PF.

Another strength of our study is the practical applicability for PE teachers. Its results highlight the vulnerability of overweight students to become unmotivated during fitness testing in PE classes. We found that this vulnerability lies in their poor PF level, thus, students who are fit, even if they are overweight, would not have that problem, which is in line with the Fat but Fit paradox [43]. Increasing overweight students′ PF would be the desired solution. It could be pursued, for example, by increasing their physical activity levels [44], which is one of the main goals in school PE. However, its feasibility is at the very least complicated for PE teachers. Another way to improve the intrinsic motivation toward fitness testing in overweight students is to improve their perceived PF. PE teachers could facilitate it through the development of motivational and methodological strategies in order to create a learning environment to promote perceptions of competence [45,46]. Some of them are recommended by Wiersman and Sherman [6] and Silverman et al. [8] for fitness testing sessions (e.g., to provide feedback focusing on controllable factors, such as effort, or to reinforce improvement, instead of normative-based feedback). We also consider that these recommendations are especially relevant in PE classes with overweight students.

## 6. Conclusions

In conclusion, our findings revealed that overweight students are less intrinsically motivated and more externally regulated and amotivated toward fitness testing in PE classes than their non-overweight classmates. In all cases, the effect of weight status on motivation during fitness testing was not a direct factor, but was mediated solely by objective PF. In addition, perceived PF proved to have a mediating role as second influence between weight status and intrinsic motivation. PE teachers should avoid repeating past mistakes, and fitness testing in PE lessons should be oriented toward health control, be compatible with educational objectives, and favour a self-determined motivation, especially in overweight students. For these purposes, PE teachers may develop motivational and methodological strategies in order to increase overweight students′ objective PF (e.g., creating opportunities for physical activity) and their perceptions of PF (e.g., evaluating their effort rather than their results).

## Figures and Tables

**Table 1:** Characteristics of the sample.

	*Mean* ± *Standard Deviation*
Age	13.42 ± 1.16
Boys (%)	55.80
Weight (kg)	56.01 ± 13.14
Height (cm)	161.79 ± 8.75
Body mass index (kg/cm^2^)	21.26 ± 4.06
Overweight (%)	31.30
20-m shuttle run (paliers)	3.19 ± 2.09
Handgrip strength (kg)	26.35 ± 7.01
Handgrip strength/weight (kg/kg)	0.47 ± 0.11
Standing long jump (cm)	161.07 ± 32.83
Global physical fitness index (z)	0.00 ± 0.83
Perceived physical fitness (1–4)	2.82 ± 0.58
Situational intrinsic motivation (1–7)	4.91 ± 1.51
Situational identified regulation (1–7)	4.87 ± 1.30
Situational external regulation (1–7)	4.48 ± 1.23
Situational amotivation (1–7)	3.55 ± 1.42

Note. Global physical fitness index = mean of the cardiorespiratory and muscular fitness z-scores, both specific by age and gender.

**Table 2:** Correlations and differences by weight status for global physical fitness index, perceived physical fitness and motivation associated with fitness testing in physical education.

							*Mean* ± *SD* by Weight Status		
	1	2	3	4	5	6	Non-Overweight	Overweight	*p*
1. Global physical fitness index (z)							0.25 ± 0.77	−0.55 ± 0.67	<0.001
2. Perceived physical fitness (1–4)	0.419 ***						2.89 ± 0.58	2.68 ± 0.55	<0.001
3. Situational intrinsic motivation (1–7)	0.283 ***	0.365 ***					5.08 ± 1.45	4.52 ± 1.55	<0.001
4. Situational identified regulation (1–7)	0.165 ***	0.299 ***	0.713 ***				5.09 ± 1.34	4.98 ± 1.34	0.165
5. Situational external regulation (1–7)	−0.114 **	−0.045	0.172 ***	0.024			4.62 ± 1.35	4.83 ± 1.26	0.010
6. Situational amotivation (1–7)	−0.199 ***	−0.164 ***	−0.433 ***	−0.359 ***	0.309 ***		3.45 ± 1.44	3.75 ± 1.40	0.033

Note. ** *p* < 0.01, *** *p* < 0.001. *SD* = standard deviation.

**Table 3:** Total, direct and indirect effects of weight status [non-overweight (reference) vs. overweight; X] on motivation associated with fitness testing in physical education (Y), mediated by global physical fitness index (M1) and perceived physical fitness (M2).

	Situational Intrinsic Motivation			Situational External Regulation			Situational Amotivation		
	*B*	*SE*	95% *CI*	*B*	*SE*	95% *CI*	*B*	*SE*	95% *CI*
Total effect	−0.428	0.098	[−0.620, −0.235]	0.238	0.082	[0.076, 0.401]	0.183	0.048	[0.093, 0.283]
Direct effects:									
1. X→Y	−0.180	0.102	[−0.382, 0.020]	0.145	0.091	[−0.034, 0.324]	0.016	0.105	[−0.191, 0.223]
2. M_1_→Y	0.187	0.064	[0.059, 0.314]	−0.139	0.057	[−0.252, −0.025]	−0.194	0.066	[−0.325, −0.063]
3. M_2_→Y	0.474	0.082	[0.311, 0.636]	0.049	0.073	[−0.095, 0.194]	−0.166	0.085	[−0.333, 0.000]
Indirect effects:									
1. X→ M_1_→Y	−0.140	0.048	[−0.242, −0.050]	0.104	0.047	[0.013, 0.198]	0.146	0.051	[0.049, 0.252]
2. X→ M_2_→Y	0.010	0.026	[−0.042, 0.062]	−0.013	0.021	[−0.064, 0.023]	−0.003	0.010	[−0.032, 0.011]
3. X→ M_1_→ M_2_→Y	−0.117	0.027	[−0.177, −0.069]	−0.012	0.018	[−0.051, 0.022]	0.041	0.023	[−0.014, 0.089]
	*R* ^2^	*F* (7, 526)	*p*	*R* ^2^	*F* (7, 526)	*p*	*R* ^2^	*F* (7, 526)	*p*
Summary model	0.193	21.061	<0.001	0.036	3.367	.002	0.048	4.520	0.002

Note. *B* = non standardised effect. *SE* = standard error. *CI* = confidence interval. The analyses were adjusted by age, gender and school. The effects were significant when the upper and lower bound of the 95% *CI* did not contain zero. a. The specific indirect effect contrast definitions did not show significant differences between the effect number 1 and 2 in this model.
